# Corticospinal effects and motor performance changes following resistance training in healthy adults: a systematic review and meta-analysis of transcranial magnetic stimulation

**DOI:** 10.3389/fphys.2026.1852484

**Published:** 2026-07-01

**Authors:** Jie Li, Pingqing Hu

**Affiliations:** China Wushu School, Beijing Sport University, Beijing, China

**Keywords:** corticospinal excitability, healthy adults, meta-analysis, motor performance, resistance training, transcranial magnetic stimulation

## Abstract

**Background:**

Resistance training is widely utilized to enhance motor performance in healthy populations. Its effects arise not only from peripheral muscular adaptations but also from neuroplastic changes within the central nervous system. Transcranial magnetic stimulation enables noninvasive quantification of corticospinal excitability; however, existing evidence in apparently healthy adults lacks systematic synthesis.

**Objective:**

This systematic review and meta-analysis aimed to evaluate the effects of resistance training on corticospinal excitability and motor performance in healthy adults.

**Methods:**

Following the PRISMA 2020 guidelines, a comprehensive search was performed in Web of Science, PubMed, Embase, Cochrane Library, SPORTDiscus, Scopus, and China National Knowledge Infrastructure databases, with the cutoff date of February 27, 2026. Randomized trials were included if they involved healthy adults, with the experimental group receiving resistance training and the control group receiving sedentary activity or no exercise intervention. Data extraction and risk-of-bias assessment using the Cochrane tool were conducted independently by two researchers. Statistical analyses were performed using R software with a random-effects model to compute standardized mean differences (SMDs) with 95% confidence intervals (95% CI), accompanied by heterogeneity testing and publication bias evaluation.

**Results:**

Eleven studies encompassing 244 healthy adults were included. Meta-analysis demonstrated that resistance training significantly increased corticospinal excitability (SMD = 0.59, 95% CI [0.10, 1.08], *p* = 0.003; I² = 69.3%) and significantly improved motor performance (SMD = 0.40, 95% CI [0.02, 0.79], *p* = 0.04; I² = 64.8%). The included studies were of high methodological quality (mean Cochrane score 6.4), and funnel plots indicated a low risk of publication bias.

**Conclusion:**

Resistance training significantly enhances corticospinal excitability and improves motor performance in healthy adults. These findings affirm resistance training as an effective modality for inducing central neural plasticity and furnish an evidence-based foundation for the development of precision training prescriptions. Future research should further elucidate the dose–response relationships between specific training parameters and neural adaptations.

## 1 Introduction

Resistance training (RT), as a safe, cost-effective, and widely accessible non-pharmacological intervention, has been extensively adopted to enhance muscular strength, explosive strength, and overall motor performance in healthy populations (Hortobágyi et al., 2021). In healthy individuals, motor performance, encompassing maximal voluntary contraction, muscular endurance, coordination, and the efficiency of skill acquisition, depends not only on peripheral muscle hypertrophy and metabolic adaptations but also on the plasticity of the central nervous system (Škarabot et al., 2021). Among these factors, cortical excitability serves as a core indicator of the responsiveness of neurons in the primary motor cortex (M1) to afferent stimuli, playing a pivotal role in motor control, force output regulation, and fatigue management (Schuler et al., 2025). Through repeated high-intensity contractions, resistance training can modulate the excitation-inhibition balance within the corticospinal tract by inducing synaptic plasticity, altering neurotransmitter balance, such as reducing GABAergic inhibition and enhancing glutamatergic excitation, and upregulating brain-derived neurotrophic factor (BDNF), thereby optimizing motor output and facilitating neural adaptations (Zhou et al., 2022).

Since its initial report by Barker et al. in 1985 (Barker et al., 1985), transcranial magnetic stimulation (TMS) has become the gold standard non-invasive technique for quantifying cortical excitability. This technique involves applying a brief magnetic field over the scalp to stimulate the motor cortex and induce motor evoked potentials (MEPs) in peripheral muscles. It enables precise measurement of parameters such as resting motor threshold (RMT), MEP amplitude, short-interval intracortical inhibition (SICI), and intracortical facilitation (ICF). These parameters reflect the state of corticospinal excitability and inhibitory networks. Compared with traditional electrophysiological methods, TMS provides high spatiotemporal resolution, noninvasiveness, and excellent repeatability. It has been widely applied in motor neuroscience, particularly for evaluating training-induced neural plasticity (Moscatelli et al., 2021).

Previous studies have demonstrated that resistance training exerts a pronounced dose-response relationship with cortical excitability. Acute resistance training significantly increases MEP amplitude and reduces SICI in healthy young adults, indicating that a state of disinhibition facilitates strength gains consolidation and motor learning. Chronic resistance training, by contrast, promotes long-term potentiation (LTP)-like plasticity through repeated activation of motor circuits. Existing evidence has shown that RT can markedly elevate corticospinal excitability, which correlates positively with gains in muscular strength, while also shortening the cortical silent period; however, its influence on SICI remains inconsistent (Gómez-Feria et al., 2023); These neurophysiological changes have been associated with improvements in motor performance, indicating a potential relationship between cortical excitability and motor function following resistance training. Both animal and human studies have reported that resistance training enhances synaptic efficacy within the motor cortex, reduces RMT, and shortens MEP latency (Kida et al., 2016; Moscatelli et al., 2016). Such adaptations appear more evident in athletes, whereas supporting evidence in the general healthy population remains comparatively limited (Turco and Nelson, 2021).

However, the existing evidence still exhibits notable limitations. First, the majority of studies have focused on athletes or specific strength disciplines, whereas randomized controlled trials targeting general healthy populations feature relatively small sample sizes, making it difficult to control for individual differences such as baseline excitability, genetics, and lifestyle factors. Second, training protocols exhibit substantial heterogeneity: dynamic versus isometric contractions, single acute sessions versus chronic interventions spanning multiple weeks, and variations in load intensity and rhythm produce inconsistent effects on TMS parameters (Siddique et al., 2020a). Some studies indicate that excitability transiently increases after acute training before returning to baseline levels, whereas chronic training produces more persistent effects; however, other reports show no significant changes or even inhibition (Latella et al., 2016). Third, the associations between changes in cortical excitability and motor performance measures are primarily derived from single studies, lacking systematic quantitative integration. This deficiency impedes the clarification of causal mechanisms and translational value (Škarabot et al., 2021). Fourth, methodological differences along with publication bias further complicate the interpretation of results (Di Virgilio et al., 2022). Although systematic reviews and meta-analyses addressing corticospinal adaptations to resistance training are available, evidence specifically targeting general healthy populations remains limited, and few studies have comprehensively integrated, relied on TMS measurements, or comprehensively integrated the effects of resistance training interventions on both corticospinal excitability and motor performance (Lockyer et al., 2021). The evidence gap is particularly pronounced in non-athlete populations. This meta-analysis systematically evaluates the overall effects of resistance training interventions on corticospinal excitability and motor performance in healthy populations. Sensitivity analyses and investigations of heterogeneity are also included. These steps provide a solid basis for optimizing strategies in health promotion and sports rehabilitation while laying the foundation for future mechanistic research on neural plasticity induced by resistance training.

## 2 Methods

The study was reported in accordance with the PRISMA guidelines (Page et al., 2021). The protocol was registered on the PROSPERO database (https://www.crd.york.ac.uk/PROSPERO/), registration number: CRD420261360071.

### 2.1 Literature search strategy

The search strategy was developed in consultation with a medical librarian experienced in systematic review database searching (Y.M.). The literature screening process was independently performed by two reviewers (J.L. and P.Q.H.), with disagreements resolved by discussion or consultation with a third reviewer (Z.Q.L) when necessary. A systematic literature search was conducted in the Web of Science, PubMed, SPORTDiscus, Scopus, Embase, Cochrane Library, and China National Knowledge Infrastructure (CNKI) databases from inception to February 27, 2026. The search strategy combined controlled vocabulary and free-text terms. Key concepts included “resistance training” OR “strength training”, “transcranial magnetic stimulation” OR “TMS” OR “motor evoked potential” OR “MEP”, “cortical excitability” OR “corticospinal excitability”, and “healthy” OR “healthy adults” OR “healthy population”, along with their synonyms and related terms. Boolean operators were applied as follows: synonyms within each concept were combined using “OR”, and different concepts were combined using “AND”, with “NOT” used where necessary to exclude irrelevant studies. The search strategy was adapted to each database with appropriate field restrictions (title, abstract, and keywords). No language restrictions were applied during the literature search. In addition, the reference lists of relevant systematic reviews and included studies were manually screened to identify any additional eligible studies.

### 2.2 Inclusion and exclusion criteria

Inclusion and exclusion criteria were predefined according to the PICOS (Population, Intervention, Comparison, Outcomes, and Study design) framework.

Inclusion criteria were as follows: (1) studies involving healthy adults (aged ≥ 18 years) without a history of neurological or musculoskeletal disorders; no restrictions were placed on sex or training status (including both untrained individuals and those with regular exercise experience); (2) the intervention group received resistance training, and studies were included only if transcranial magnetic stimulation (TMS) data were extractable; no restrictions were imposed on training duration or frequency; (3) the control group consisted of sedentary individuals or those receiving no exercise intervention; (4) outcomes included measures of cortical excitability and motor performance; and (5) randomized controlled trials (RCTs) including crossover designs.

Exclusion criteria were as follows: (1) studies involving patients, rehabilitation populations, athletes, unhealthy adults, or animal models; (2) studies that did not clearly describe the exercise intervention protocol or that employed non-exercise interventions (e.g., pharmacological or physical therapies); (3) studies that did not report quantitative outcomes related to cortical excitability or motor performance; and (4) non-randomized studies (e.g., cross-sectional or observational studies), qualitative studies, conference abstracts, reviews, book chapters, or studies with incomplete data from which relevant outcomes could not be extracted.

### 2.3 Data extraction

Data extraction was performed independently by two reviewers (J.L. and P.Q.H.) using a standardized, pre-piloted electronic form designed in accordance with the PICOS framework. Discrepancies are resolved by discussion and by consultation of a third reviewer, if needed (Z.Q.L). The following data were extracted from each included study: (1) study characteristics (first author, publication year, study design); (2) participant information (total sample size, number per group, mean age, and eligibility confirmation of healthy adults aged ≥18 years with no neurological or musculoskeletal disorders); (3) intervention and control details (resistance training protocol, including exercise type, frequency, and duration; control condition); (4) TMS outcome measures of corticospinal excitability; and (5) motor performance outcomes. When data were missing or not explicitly reported, corresponding authors were contacted to obtain the original values.

### 2.4 Risk of bias assessment

Two reviewers independently assessed the risk of bias for the included studies according to the seven domains specified in the Cochrane Handbook for Systematic Reviews of Interventions: random sequence generation, allocation concealment, blinding of participants and personnel, blinding of outcome assessment, incomplete outcome data, selective reporting, and other bias. Discrepancies were resolved through discussion with a third reviewer until consensus was reached. The final assessment results were used to generate risk-of-bias graphs using Review Manager 5.4 software for intuitive visual presentation of the quality of the included studies.

### 2.5 Statistical analysis

Statistical analyses of the included studies were performed using R software. Standardized mean differences (SMDs) were used as effect sizes and interpreted as follows: < 0.20, negligible; 0.20–0.49, small; 0.50–0.79, medium; ≥ 0.80, large (Cohen et al., 2013). Heterogeneity among studies was quantified using I², ranging from 0% to 100%. A random-effects model was applied when I² > 50%, otherwise a fixed-effects model was used (Barili et al., 2018). Statistical significance was set at *p* < 0.05. Sensitivity analyses were conducted using a leave-one-out approach to assess the robustness of the findings (Viechtbauer and Cheung, 2010). Due to the insufficient number of studies included in the meta-analysis, neither subgroup analyses nor meta-regression analyses were performed.

## 3 Results

### 3.1 Literature search

**Figure 1:**
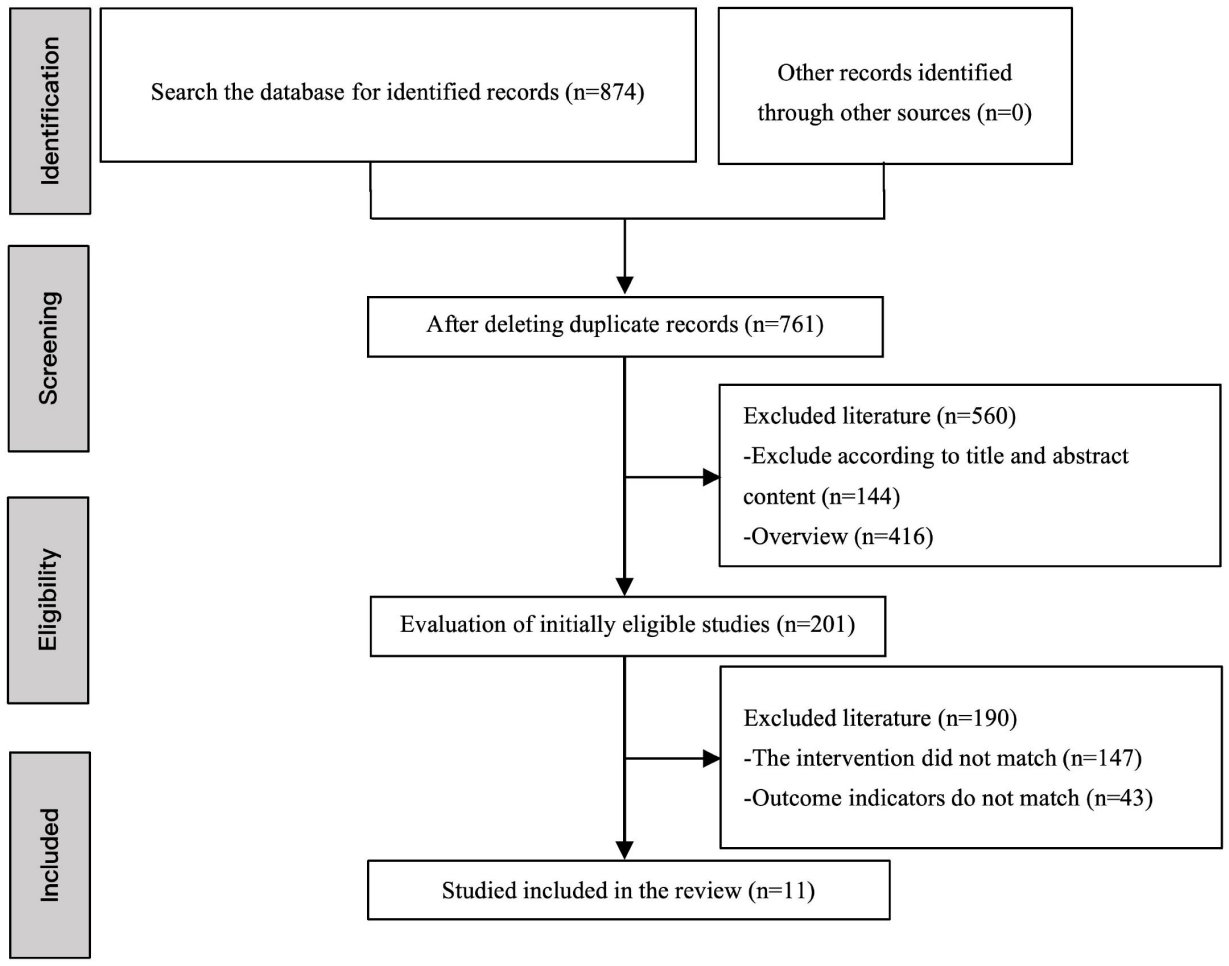
The PRISMA flow chart of study selection.

A total of 874 records were retrieved based on the initial search strategy. All identified records were imported into EndNote 21 software, where 113 duplicate records were removed, leaving 761 unique citations. Subsequently, an initial screening of titles was performed, which led to the exclusion of 416 records identified as either irrelevant to the research topic or review articles. Following abstract screening, a further 144 records were excluded, leaving 201 studies for full-text evaluation. These remaining studies underwent a comprehensive full-text review, resulting in the exclusion of 190 studies due to reasons such as non-randomized controlled trial designs, lack of relevant outcome measures, non-exercise interventions, or study populations comprising unhealthy adults. Ultimately, 11 studies were included in the meta-analysis. The detailed literature screening process is illustrated in **Figure 1**.

### 3.2 Characteristics of the included studies

The characteristics of the included studies are summarized in **Table 1**. The 11 included studies comprised 9 randomized controlled trials (RCTs) and 2 randomized crossover trials. The study populations across all trials comprised healthy adults without a history of neurological or musculoskeletal disorders. These studies were published between 2005 and 2020, with a cumulative sample size of 244 participants. Due to participant attrition in some studies or the repeated accounting of subjects in crossover designs, the values presented in **Table 1** represent the effective sample sizes specifically included in the meta-analysis. Interventions for the experimental groups consisted of various resistance training protocols, while control groups typically involved no exercise, sitting quietly, or maintaining habitual physical activity levels. The primary outcome measures focused on indices of cortical excitability, with motor performance serving as the secondary outcome.

**Table 1:** Characteristics of the included studies.

Study	Design	Sample size(T/C)	Age	TMS parameters	Intervention	Intervention frequency	Control group	Outcomes
Siddique 2020 (Siddique et al., 2020b)	RCT	42(32/10)	25.1 ± 5.8	Biceps brachii,primary motor cortex, 130% AMT, 80% AMT, 3 ms ISI	Progressive strength training	4 weeks	No training	One-repetition maximum; short-interval intracortical inhibition
Mason 2020 (Mason et al., 2020)	RCT	18(9/9)	23.45 ± 4.2	Flexor Carpi Radialis, primary motor cortex,130/150/170% AMT; 80/120% AMT, 3/10/100 ms ISI	High-intensity wrist flexor training	2 weeks, 6 times per week	Sit quietly	one-repetition Maximum strength; short-interval intracortical inhibition
Mason 2019 (Mason et al., 2019)	cross−over design	18(18/18)	females:23.38 ± 3.29 males:26.8 ± 9.6	Flexor carpi radialis, Extensor carpi radialis,primary motor cortex,130/150/170% AMT; 80/120% AMT, 3/10/100 ms ISI	Single high-load wrist flexor training	Acute intervention: 4 sets, 6–8 repetitions per set, 2.5 min rest between sets	Sit quietly	One-repetition maximum of the synergist muscles; short-interval intracortical inhibition
Latella 2017 (Latella et al., 2017)	cross−over design	14(14/14)	26.2 ± 3.1	Rectus femoris, Primary motor cortex, 120% AMT; 90/120% RMT,3/12 ms ISI;120/120% RMT,100 ms ISI	High-load strength training and muscle hypertrophy training	Acute intervention	Sit quietly after a 5-minute warm-up on a stationary bike	Maximal voluntary isometric contraction; motor-evoked potential amplitude
Leung 2017 (Leung et al., 2017)	RCT	33(22/11)	26.1 ± 6.8	Right biceps brachii, Primary Motor Cortex, 10~40% AMT; 80% AMT/1mV MEP	Dumbbell curls with the dominant arm	4 weeks, 3 times per week	daily physical activity	Maximal voluntary dynamic strength; motor evoked potentials
Nuzzo 2017 (Nuzzo et al., 2017)	RCT	21(10/11)	T:23.5 ± 7.5 C:23.0 ± 4.2	Right biceps brachii, Primary Motor Cortex, 45–100% stimulator output	High-intensity isometric contraction of the elbow flexors	4 weeks, 12 training sessions total	Sit quietly	Maximal strength; voluntary cortical activation
Kidgell 2015 (Kidgell et al., 2015)	RCT	27(18/9)	26.0 ± 1.5	Flexor Carpi Radialis, Primary Motor Cortex, 90/110/130/150/170/190/210/230% AMT; 80/120% AMT, 3 ms ISI	Maximum concentric contraction and maximum eccentric contraction of the right wrist flexors	4 weeks, 3 times per week, 12 sessions total	No training	Muscle thickness; short-interval Intracortical inhibition
Goodwill 2012 (Goodwill et al., 2012)	RCT	14(7/7)	18-35	Rectus femoris, Primary motor cortex, 70/120% AMT; 3 ms ISI	Unilateral strength training of the dominant right leg	3 weeks, 3 times per week, 9 sessions total	No training	Short-interval intracortical inhibition
Kidgell 2010 (Kidgell and Pearce, 2010)	RCT	16(8/8)	24.12 ± 5.21	right first dorsal interosseous, Primary motor cortex, 110% AMT	Index finger abduction contraction	4 weeks, 10 training sessions total	No training	maximal voluntary contraction; motor-evoked potential amplitude
Carroll 2009 (Carroll et al., 2009)	RCT	17(8/9)	19-35	wrist muscles, Primary motor cortex, 0.85–2.0 × RMT, 5–8 distinct stimulus intensities	Dynamic resistance training for right wrist radial deviator muscles	4 weeks, 3 times per week, 12 sessions total	No training	motor evoked potentials
Jensen 2005 (Jensen et al., 2005)	RCT	24(16/8)	25 ± 5	Biceps brachii, Triceps brachii,Primary motor cortex, 0.6–2.0 × MEP threshold, 10–15 distinct stimulus intensities	Strength training (high-load training of the right arm biceps) and motor skill training (skill training of the right arm elbow flexors)	4 weeks, 3 times per week	No training	average maximal dynamic strength at a single maximal repetition load

T, Treatment group; C, Control group; AMT, Active Motor Threshold; RMT, Resting Motor Threshold; ISI, Inter-Stimulus Interval; MEP, Motor Evoked Potential.

### 3.3 Literature quality assessment

**Figure 2:**
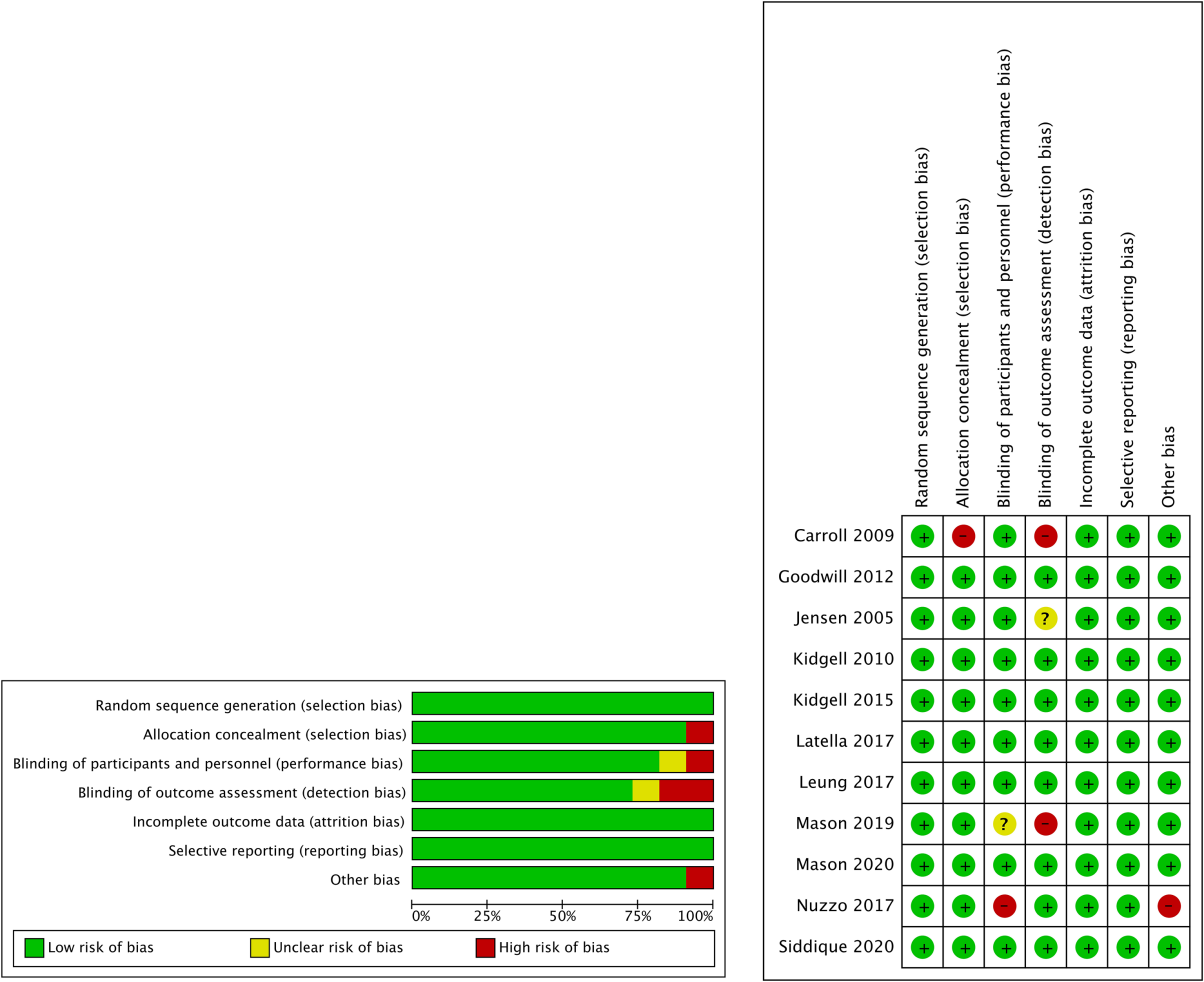
Risk of bias graph.

Risk of bias assessment using the Cochrane Risk of Bias tool is summarized in **Figure 2** and **Table 2**. Among the included studies, seven studies were rated as low risk across all assessed domains. One study showed high risk in at least one domain, while three studies had unclear risk in one or more domains.

**Table 2:** Risk of bias assessment of included studies.

Study	①	②	③	④	⑤	⑥	⑦	Score
Siddique 2020 (Siddique et al., 2020b)	+	+	+	+	+	+	+	7
Mason 2020 (Mason et al., 2020)	+	+	+	+	+	+	+	7
Mason 2019 (Mason et al., 2019)	+	+	?	–	+	+	+	5
Nuzzo 2017 (Nuzzo et al., 2017)	+	+	–	+	+	+	–	5
Latella 2017 (Latella et al., 2017)	+	+	+	+	+	+	+	7
Leung 2017 (Leung et al., 2017)	+	+	+	+	+	+	+	7
Kidgell 2015 (Kidgell et al., 2015)	+	+	+	+	+	+	+	7
Goodwill 2012 (Goodwill et al., 2012)	+	+	+	+	+	+	+	7
Kidgell 2010 (Kidgell and Pearce, 2010)	+	+	+	+	+	+	+	7
Carroll 2009 (Carroll et al., 2009)	+	–	+	–	+	+	+	5
Jensen 2005 (Jensen et al., 2005)	+	+	+	–	+	+	+	6

①Random sequence generation; ②Allocation concealment; ③Blinding of participants and personnel; ④Blinding of outcome assessment; ⑤Incomplete outcome data; ⑥Selective reporting; ⑦Other bias.

### 3.4 Meta-analysis results

#### 3.4.1 Cortical excitability analysis

**Figure 3:**
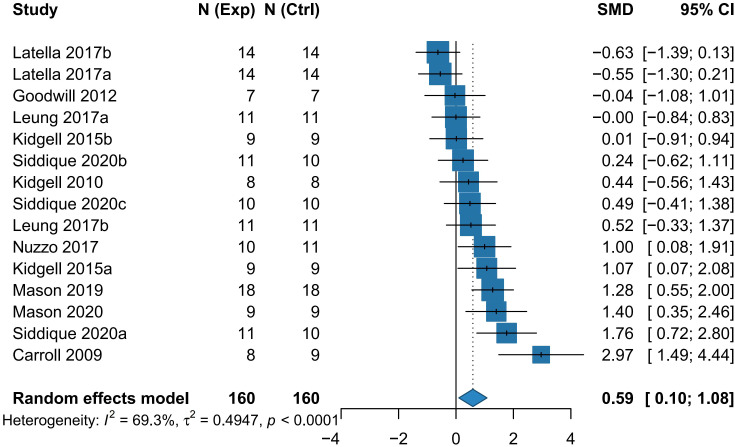
Forest plot of the pooled effects of exercise interventions on cortical excitability.

The forest plot (**Figure 3**) illustrated the pooled effect of RT interventions on cortical excitability by synthesizing 15 independent effect-size estimates extracted from the included studies. Heterogeneity testing indicated between-study heterogeneity (I² = 69.3%, *p* < 0.0001); accordingly, a random-effects model was applied. The analysis revealed that RT interventions produced a statistically significant positive effect on cortical excitability (SMD = 0.59, 95% CI [0.10, 1.08], *p* = 0.003), with effect sizes ranging from small to large.

#### 3.4.2 Motor performance analysis

**Figure 4:**
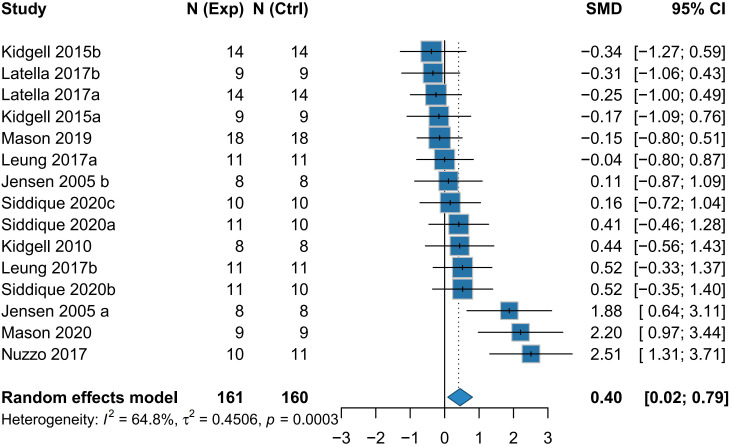
Forest plot of the pooled effects of exercise interventions on motor performance.

The forest plot (**Figure 4**) illustrated the pooled effect of RT interventions on motor performance by synthesizing 15 independent effect-size estimates extracted from the included studies. Heterogeneity testing indicated substantial between-study heterogeneity (I² = 64.8%, *p* = 0.0003); accordingly, a random-effects model was applied. The analysis revealed that participants in the experimental group exhibited significantly superior motor performance compared to the control group [SMD = 0.40, 95% CI (0.02, 0.79), *p* = 0.04], with effect sizes ranging from small to moderate.

### 3.5 Publication bias

**Figure 5:**
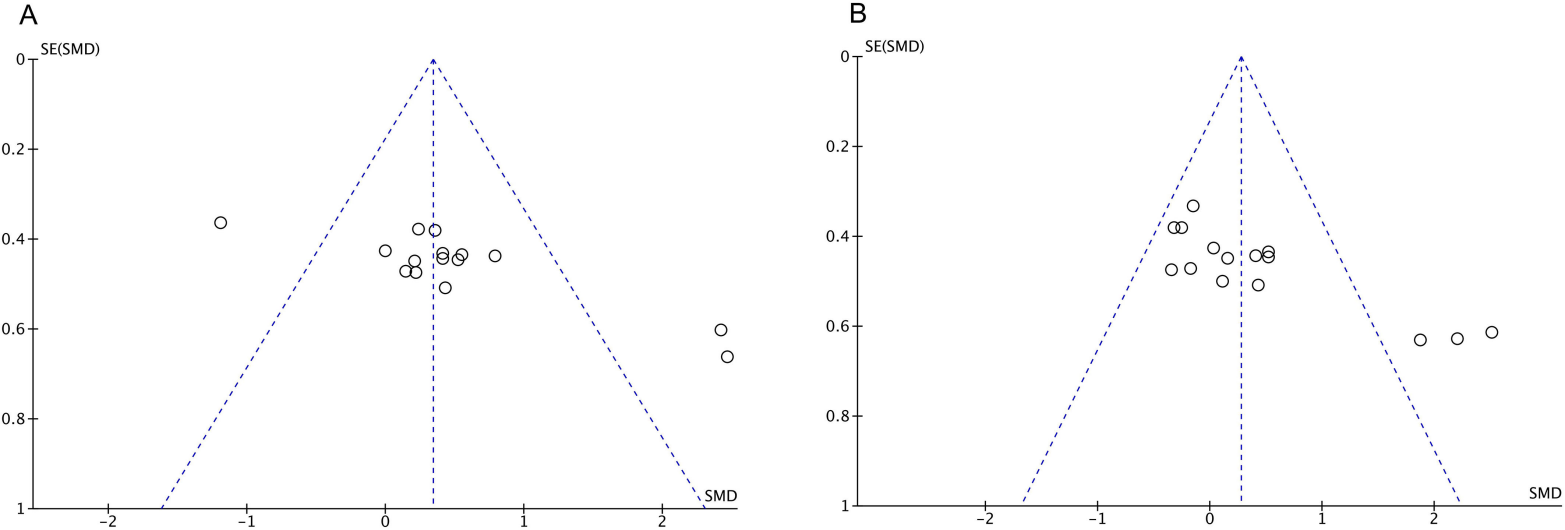
Funnel plots of included literature: **(A)** cortical excitability funnel plot; **(B)** motor performance funnel plot.

The funnel plot was used to assess potential publication bias among the included studies. Although a minority of data points were located at the edges, potentially indicating small-sample effects, the overall distribution appeared relatively symmetrical (**Figure 5**). This finding suggests no obvious evidence of publication bias. However, given the limited number of included studies, the ability of the funnel plot to reliably detect asymmetry is constrained, and the results should therefore be interpreted with caution.

### 3.6 Sensitivity analysis

**Figure 6:**
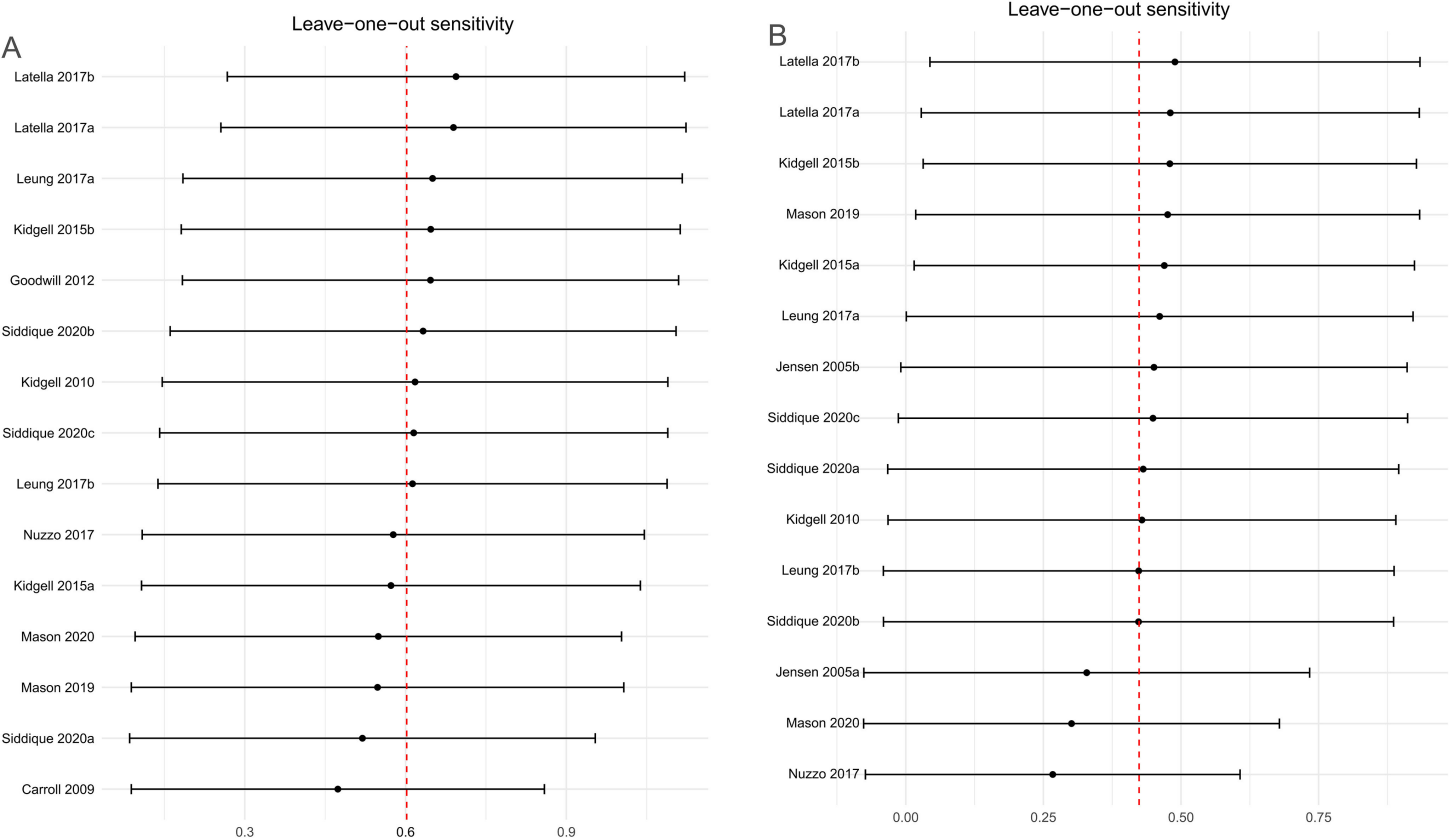
Sensitivity analysis. **(A)** cortical excitability sensitivity analysis; **(B)** motor performance sensitivity analysis.

Sensitivity analysis was performed using a leave-one-out approach, in which each included study was sequentially excluded from the meta-analysis pool (**Figure 6**). The results demonstrated that the pooled effect size remained statistically significant and consistent in both direction and magnitude across all iterations. This stability confirms the robustness and reliability of the meta-analytic conclusions regarding the positive effects of exercise interventions.

## 4 Discussion

This meta-analysis represents the first comprehensive systematic review and meta-analysis of the impact of RT on corticospinal excitability and motor performance in healthy adults. Based on the 11included RCTs and randomized crossover trials, a total of 15 independent effect-size estimates were extracted for analysis. The results demonstrate that RT interventions significantly enhance both cortical excitability and motor performance, despite the presence of high inter-study heterogeneity. The symmetrical morphology of the funnel plots suggests a low risk of publication bias within this meta-analysis. Furthermore, the included studies achieved an average Cochrane risk-of-bias score of 6.4, indicating high overall methodological quality. These findings provide quantitative evidence for central neural plasticity induced by resistance training and address a significant gap in the literature by integrating TMS indices and motor performance data specifically for the general healthy population.

The present study demonstrates that RT significantly enhances cortical excitability, a finding that aligns with previous evidence regarding RT-induced LTP-like plasticity (Akalu et al., 2025; Liang and Liu, 2025). Due to the inherent task specificity resistance training, the central nervous system must consolidate neural resources to achieve high-intensity drive; consequently, the induced cortical activity exhibits greater focus and reproducibility. By means of repeated high-intensity muscular contractions, RT facilitates excitatory synaptic transmission within the primary M1, characterized by a reduction in gamma-aminobutyric acid (GABA)-mediated SICI, an intensification of glutamatergic excitation, and the upregulation of BDNF expression (Cefis et al., 2023; Lecce et al., 2026). Furthermore, RT exerts a more potent neural drive on the corticospinal pathway. High-load resistance training can significantly activate the M1 and associated subcortical structures within a Narrow temporal window, leading to elevated discharge frequencies and synchronized outputs among cortical neurons (Takarada, 2025). This robust neuroexcitatory stimulation is considered capable of inducing synaptic plasticity responses akin to long-term potentiation, thereby optimizing the efficiency of cortical efferent signaling and enhancing neuromuscular recruitment capabilities (Tanel et al., 2025; Zhang et al., 2025).

RT exhibits greater adaptability in neuromuscular synergistic control. Through repeated high-intensity contractions, resistance training prompts the nervous system to optimize motor unit recruitment sequences and discharge patterns, thereby enhancing the reaction speed and output synchronization of motor neurons. This “neural drive enhancement effect” not only directly augments muscular strength but also indirectly facilitates motor performance by increasing cortical excitability (Rong et al., 2025; Del Vecchio et al., 2026). Previous studies have demonstrated that unilateral resistance training can induce enhanced corticospinal excitability in the contralateral limb, suggesting that the neural adaptations to resistance training possess cross-education and systemic characteristics (Kidgell et al., 2011). Furthermore, the high mechanical loading inherent in resistance training provides potent dual stimulation from both sensory feedback and motor commands. During high-intensity resistance training, muscles, tendon organs, and proprioceptors continuously transmit a vast array of sensory signals to the central nervous system. These signals form a closed-loop feedback mechanism with motor commands, further reinforcing the plasticity of the motor cortex (Feulner et al., 2025). This “sensory-motor coupling” process is considered a fundamental neurological basis for heightened cortical excitability (Peng et al., 2025). Consequently, the specificity of the training task and its load characteristics remain pivotal factors influencing cortical excitability.

It is noteworthy that the majority of intervention protocols included in this study involved short-term training of approximately four weeks, typically with a frequency of three sessions per week. This suggests that even relatively brief periods of resistance training are sufficient to induce measurable central neural adaptations. However, the high degree of inter-study heterogeneity indicates significant variability in effect sizes across the literature. This divergence likely stems from confounding factors such as training modality, load intensity, tempo control, target muscle groups, and specific TMS parameters, including stimulation intensity, coil type, and the methodology of MEP recording. Therefore, although some evidence suggests that resistance training may reduce intracortical inhibition, the current evidence does not support a definitive conclusion regarding the direction or magnitude of SICI changes. Future studies using standardized paired-pulse TMS protocols and larger sample sizes are needed to clarify whether SICI is consistently modulated by resistance training.

This study has several limitations that should be considered when interpreting the findings. First, the number of included studies was limited, with a total cumulative sample size of 244 participants. Several individual studies included fewer than 20 participants, making the results more susceptible to small-study effects and individual variability in baseline corticospinal excitability, which may have contributed to the observed heterogeneity. Second, most existing studies have focused on single-joint upper-limb muscle groups (e.g., wrist flexors, biceps brachii, and index finger abductors), whereas evidence regarding lower-limb muscles and multi-joint compound exercises remains limited. This restricts the generalizability of the findings to more complex and systemic resistance training modalities, such as squats and deadlifts. Third, substantial methodological variability across studies, including differences in training protocols and TMS assessment parameters, together with the limited number of available studies, precluded sufficiently powered subgroup analyses. Consequently, it remains difficult to identify the optimal resistance training prescription for promoting neuroplastic adaptations. Both acute and chronic studies were included, which may have contributed to heterogeneity and limits causal interpretation of the findings.

## 5 Conclusion

This systematic review and meta-analysis suggests that resistance training may be associated with increased corticospinal excitability and improved motor performance in healthy adults. These findings support the possibility that resistance training contributes not only to peripheral muscular adaptations but also to neurophysiological changes within the central nervous system. However, the results should be interpreted with caution due to the limited number of included studies, relatively small sample sizes, methodological heterogeneity, and variations in training protocols and TMS parameters. Although the overall findings were generally consistent across studies, the observed heterogeneity indicates that the magnitude and nature of training-induced neuroplastic adaptations may depend on specific intervention and assessment characteristics. Furthermore, while visual inspection of the funnel plot did not indicate substantial asymmetry, the small number of included studies limits the ability to reliably assess publication bias. Taken together, the current evidence suggests that resistance training may represent a promising non-pharmacological approach for promoting neuroplasticity and enhancing motor function in healthy adults. Nevertheless, further high-quality studies with larger sample sizes, standardized TMS methodologies, and more comparable training protocols are needed to strengthen the evidence base, clarify dose–response relationships, and identify factors that influence individual responsiveness to training.

## Data Availability

The original contributions presented in the study are included in the article/supplementary material. Further inquiries can be directed to the corresponding author.
